# Effectiveness of Technology-Based Interventions in Promoting Lung Cancer Screening Uptake and Decision-Making Among Patients

**DOI:** 10.3390/ijerph22081250

**Published:** 2025-08-09

**Authors:** Safa Elkefi, Nelson Gaillard, Rongyi Wu

**Affiliations:** 1 School of Systems Science and Industrial Engineering, Watson College, Binghamton University, Vestal, NY 13902, USA; 2 School of Nursing, Columbia University, New York, NY 10032, USA; 3 Mailman School of Public Health, Columbia University, New York, NY 10032, USA; 4 Emily Couric Comprehensive Cancer Center, University of Virginia, Charlottesville, VA 22903, USA

**Keywords:** lung health, lung cancer, screening, decision-making, digital health

## Abstract

This study reviews how technology-based interventions have been designed and implemented to promote lung cancer screening (LCS), support shared decision-making, and enhance patient engagement. A systematic search of six databases in February 2025 identified 28 eligible studies published between 2014 and 2025. Most interventions were home-based and self-guided, including videos, websites, mobile apps, telehealth, and patient portal messages. Common features included risk calculators, multimedia content, simplified navigation, and integration with electronic medical records. These tools aim to raise awareness, improve informed decision-making, and support smoking cessation. While 82% of studies reported positive effects on knowledge and decision-making confidence, only some showed an increased screening uptake. Key barriers included limited internet access, low digital literacy, provider time constraints, fear or anxiety, and concerns about radiation or cost. Despite these challenges, digital tools show promise in advancing LCS promotion. Their effectiveness, however, depends on thoughtful design, integration into clinical workflows, and equitable access. Future work should address structural and contextual challenges to scale digital health solutions and reduce disparities in screening participation. This review identifies both the potential and limitations of current interventions and offers guidance for enhancing impact through targeted, accessible, and user-informed approaches.

## 1. Introduction

Lung cancer (LC) remains the leading cause of cancer-related deaths, with a significant number of cases diagnosed at advanced stages [1]. According to the International Agency for Research on Cancer (IARC), approximately 2.5 million new cases of lung cancer were diagnosed worldwide in 2022, accounting for around 12% of all newly diagnosed cancers [1,2]. Additionally, LC remains the leading cause of cancer-related deaths in the world, with 1.8 million deaths recorded in 2022, constituting 19% of all cancer deaths globally [1,2].

The early detection of lung cancer through screening programs can potentially reduce mortality rates and improve patient outcomes [3,4]. According to a study led by Mount Sinai researchers, results demonstrated a 10-year lung cancer-specific survival rate of 81% (95% CI, 79–84%) and a 20-year lung cancer-specific survival rate of 81% (95% CI, 78–83%) in patients who were diagnosed with lung cancer through early CT screening [5,6]. Among patients who were diagnosed with stage I disease (≤10 mm), the survival rate was 95% (95% CI, 91–98%) [6,7]. Despite its potential in detecting LC in treatable stages, the uptake of screening remains suboptimal, especially among high-risk populations [8]. This low participation rate has been explained by many factors, including limited access to screening clinics and facilities, lack of awareness about eligibility and screening benefits, socioeconomic factors, and misconceptions about benefits and risks [9,10,11].

Recent advancements in technology present promising opportunities to mitigate existing barriers in healthcare delivery, particularly in the realm of cancer screening [12]. Technology-based interventions, including mobile health applications (mHealth), telemedicine consultations, and digital decision aids, have the potential to enhance patient education, facilitate SDM, and improve access to screening services [13]. These tools can personalize communication, offer real-time support, and empower patients to make informed decisions regarding cancer screening [14,15,16]. For instance, digital decision aids have demonstrated a significant increase in colorectal cancer screening uptake compared to tailored educational interventions and standard care practices [17,18]. Similarly, mobile health applications have been shown to effectively encourage the utilization of genetic counseling among women diagnosed with ovarian cancer [19]. Despite these documented successes in various types of cancer, there remains a noticeable scarcity of research specifically investigating the impact of technology-based interventions on lung cancer screening uptake and associated decision-making processes. Most existing studies tend to concentrate on general cancer screening or focus on particular populations or technologies [20,21], thereby highlighting a gap in understanding the broader applicability and efficacy of these interventions within the context of lung cancer screening.

To fill this gap, this study explores the effectiveness of technology-based interventions in promoting lung cancer screening uptake and enhancing patient decision-making. By assessing the impact of these interventions, we seek to identify strategies that can be implemented to increase screening participation and ultimately reduce lung cancer mortality.

## 2. Materials and Methods

**Figure 1:**
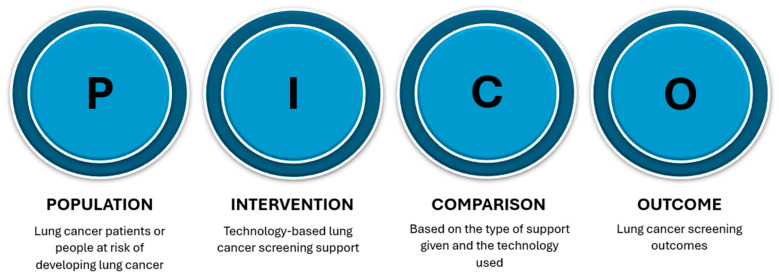
Review study design (PICO framework).

We systematically reviewed the literature according to the Preferred Reporting Items for Systematic Reviews and Meta-Analysis (PRISMA) guidelines. The review was registered on the Open Science Framework (DOI: 10.17605/OSF.IO/EQ4DX). On 23 February 2025, we searched the following databases for articles that fit our scope: ProQuest CENTRAL, Scopus, ScienceDirect, PubMed, Web of Science, and IEEE Xplore. The review was conducted following the PICO framework, as shown in Figure 1.

### 2.1. Search Strategy

We used keywords related to lung cancer, screening, and technology, as summarized in Appendix A (Table A1). We combined these terms using the Boolean operators “AND/OR” to identify the relevant studies to the scope of the review through MeSH terms such as (“lung cancer” OR “LUNG-CANCER”) AND (screening OR diagnosis OR LDCT OR “low-dose CT scan” OR detection) AND (informatic OR technology OR computer OR mhealth OR telemedicine OR telehealth OR HER OR Ehealth OR e-health OR internet OR digital OR AI OR “artificial intelligence” OR “LLM”). These search words were identified by an initial literature review and then modified by feedback from content experts and the librarian. We exported the records retrieved to EndNote 20.1 (Hoboken, NJ, USA) for duplication removal and selection processes. Additional articles were identified using the reference snowballing method from reference lists of relevant articles and systematic reviews and from select lung cancer and informatics journals.

### 2.2. Inclusion and Exclusion Criteria

Eligible papers present qualitative and/or quantitative empirical evidence, including surveys, interviews, experiments, and observational studies on technology-based interventions to support lung cancer screening among patients. We included only peer-reviewed publications written in English. We excluded opinion papers, editorials, commentaries, and articles discussing the prediction of lung cancer screening through artificial intelligence and machine learning.

### 2.3. Screening and Selection Process

The publications identified through the comprehensive search strategy were first screened by reviewing the titles and abstracts to determine their relevance to the research question and to remove any duplicates. First, an abstract and title screening was conducted. Following the title and abstract screening, the full texts of the remaining papers were obtained and read in detail to make the final selection of studies for inclusion in the systematic review. A predefined set of inclusion and exclusion criteria was applied during the full-text review to determine the eligibility of each study. A data abstraction form, developed based on the research question and objectives of the review, was used to record standardized information from each included paper. The data abstraction form was pilot-tested and refined as necessary to ensure accurate and consistent data collection. The form is attached in Appendix B (Table A2) to enhance the transparency and reproducibility of the systematic review.

### 2.4. Quality Assessment and Risk of Bias

To ensure high-quality reporting, all selection steps were agreed upon by all authors. We assessed the quality of the selected studies using the Mixed-Methods Appraisal Tool (MMAT), which evaluates qualitative, quantitative, and mixed-methods studies based on methodological rigor. For quantitative studies, we evaluated study design, sample representativeness, measurement validity, and statistical analyses, distinguishing between randomized and nonrandomized clinical trials. For mixed-methods studies, we assessed the integration of qualitative and quantitative components, including whether the study design appropriately aligned with its research questions and how data from different methodologies were synthesized. Reviewers independently assigned a risk-of-bias judgment to each study, categorizing it as low risk, high risk, or unclear risk for each criterion. Based on these assessments, studies meeting all criteria with a low likelihood of bias received a quality grade of “A”, studies partially meeting criteria with a moderate risk of bias received a quality grade of “B”, and studies failing to meet criteria with a high likelihood of bias received a quality grade of “C”. Only studies with an overall quality grade of A or B were included, while those graded C were excluded from further analysis. This structured approach ensured a rigorous evaluation of study quality and minimized potential biases in our synthesis.

## 3. Results

A total of 23,920 articles were retrieved from the following databases: ProQuest CENTRAL, Scopus, ScienceDirect, PubMed, Web of Science, and IEEE Xplore. After 10,340 duplicates were removed, we included 9856 articles for the screening. We screened the titles and abstracts of the studies and found 35 eligible articles.

**Figure 2:**
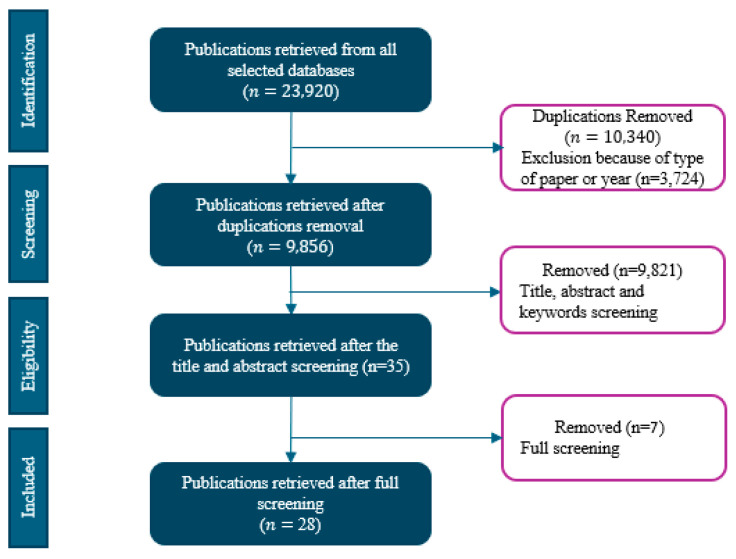
PRISMA flowchart.

Five additional articles were identified using the reference snowballing method from reference lists of relevant articles, systematic reviews, and select lung cancer and informatics journals. After a full article review, we deemed 28 articles eligible for data extraction, shown in Figure 2 below. Included studies were published between 2014 and 2025 [22,23,24,25,26,27,28,29,30,31,32,33,34,35,36,37,38,39,40,41,42,43,44,45,46,47,48,49], with 11 studies published before 2020 [22,23,24,30,31,34,38,39,40,43,47] and 17 studies published after 2020 [25,26,27,28,29,32,33,35,36,37,41,42,44,45,46,48,49]. Appendix C (Table A3) summarizes the study findings.

**Figure 3:**
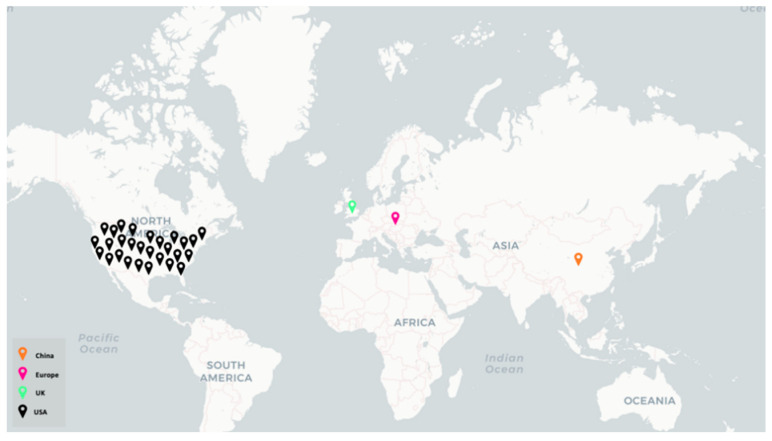
Distribution of geographic location of publication of extracted articles.

Of the included studies, 26 were based in the United States [22,23,24,25,27,28,29,30,31,32,33,34,35,36,37,38,39,40,41,42,44,45,46,47,48,49], as shown in Figure 3, 1 was based in Europe (United Kingdom [43]), and 1 was based in China [26]. In addition, two studies were designed specifically for urban populations [36,37], whereas the remainder (n = 28) did not specify whether the study was designed specifically for urban or rural communities [22,23,24,25,26,27,28,29,30,31,32,33,34,35,38,39,40,41,42,43,44,45,46,47,48,49].

The implemented interventions used a variety of technology methods, including websites (n = 4) [22,27,36,37], videos (n = 10) [23,24,32,33,34,35,40,43,45,47], telehealth appointments (n = 2) [25,48], mobile applications (n = 2) [29,44], telephone calls (n = 1) [31], and electronic patient portal messages (n = 5) [28,30,38,39,49]. The remaining interventions (n = 4) consisted of digital surveys and web-based decision aids whose type of technology was not specified [5,20,21,25].

Most interventions were delivered by research staff at large research institutions (n = 25) [22,23,24,25,26,27,28,29,30,31,32,33,34,35,36,37,38,39,40,41,42,45,46,47,49]. Others were delivered by health care providers (n = 1) [43] and trained decision counselors (n = 2) [44,48]. These interventions were delivered in different settings: at home (n = 21) [22,25,26,27,28,31,32,33,35,36,37,38,39,40,41,42,43,44,45,47,48], in clinics (n = 4) [23,24,34,46], and in both settings (n = 3) [29,30,49]. For the 10 video-based interventions, 9 reported the length of the videos [23,24,32,33,34,35,40,43,45]. For the nine videos that reported intervention length, the average length per video was 9.2 min, with videos ranging from 3.5 min [45] to 30 min [32]. For non-video, non-EHR interventions (including website, mobile applications, digital surveys, and web-based decision aids) [22,25,26,27,28,29,30,31,36,37,38,39,41,42,44,46,47,48,49], the average length of completion was 11.28 min, with completion and browsing times ranging from 5.95 min [46] to 15 min [48]. Most interventions (n = 26) were only administered once [22,23,24,25,27,28,29,30,31,32,33,34,35,36,37,38,39,40,41,42,43,44,45,46,47,48]. The remaining two interventions were implemented over time [26,49]: Zhang et al. assessed participants’ passive exposure to online health information and their likelihood to screen for lung cancer [26]; Kukhareva et al. provided a clinician-facing intervention during one period of the study and a patient-facing intervention during the second period of the study [49].

### 3.1. Study Population

Study participants were identified and recruited from a few different sources: smoking quitline or treatment programs (n = 6) [23,27,31,33,40,47], electronic medical record (EMR) data (n = 11) [28,29,30,32,34,38,39,43,44,48,49], social media (n = 2) [35,42], referred by providers (n = 2) [24,37], and others (n = 7) (including volunteers [22], VA Corporate Data Warehouse [41], in-person flyering and recruitment [36], online panel [45], telephone [25], and online survey [26]). All studies (n = 28) included participant populations comprised of current or former smokers [22,23,24,25,26,27,28,29,30,31,32,33,34,35,36,37,38,39,40,41,42,43,44,45,46,47,48,49]. Fifteen articles reported pack-year smoking history ranging from 24.08 pack-years [22] to 60.3 pack-years [27], with a mean of 44.26 pack-years [22,23,24,27,31,33,34,40,41,42,43,44,45,47,48].

### 3.2. The Technology-Based Tools Developed to Support Lung Cancer Screening Promotion

Most tools described in the included studies were developed by researchers for the study (n = 21) [22,23,24,25,26,27,28,29,30,31,32,33,34,35,36,38,39,40,41,43,47,49]. Six of the tools were previously designed or commercially available [37,42,44,45,46,48]. Of the 30 technologies developed and studied, 4 were websites [22,27,36,37], 10 were videos [23,24,32,33,34,35,40,43,45,47], 2 were telehealth appointments [25,48], 2 were mobile applications [29,44], 5 were electronic patient portal messages [28,30,38,39,49], and 1 was a telephone call [31]. The remaining interventions consisted of digital surveys and web-based decision aids [5,20,21,25].

These technologies were mostly designed for participants to be able to engage at home without having to come into a clinic (n = 21) [22,25,26,27,28,31,32,33,35,36,37,38,39,40,41,42,43,44,45,47,48]. On the other hand, four studies described technologies that were meant to be used in a clinical setting [23,24,34,46]. Three studies covered technologies that were tested at home and in clinic-based settings [29,30,49]. The technologies were mostly designed for personal use (n = 25) [22,23,24,26,27,28,29,30,31,32,33,35,36,37,38,39,40,41,42,45,46,47,49] with the remainder designed for assisted use (n = 5) [25,34,43,44,48].

#### 3.2.1. Features of Technology

The articles discussed various features of lung cancer screening promotion tools, such as information about lung cancer screening (its risks and benefits), personalization to participants’ risks and needs, health literacy support, accessibility, interactivity, and integration with electronic medical records. In terms of information and education, many tools outlined screening eligibility, risk factors, benefits, and harms of lung cancer screening. Volk et al., for example, supplemented information about lung cancer screening eligibility and risk factors with a video of a patient undergoing LDCT, a creative method to provide patients with a depiction of what LDCT is truly like [33]. Personalization of the lung cancer screening promotion tools was another key component described in the literature. Webster et al. included a pack-year calculator that allowed participants to calculate their own risk of lung cancer [27].

Some tools contained accessibility-focused features for populations with low health and digital literacy [23,24,26,27,28,29,37,42,43,45,46]. These features included read-aloud functions, simplified interfaces, and multimedia explanations, as seen in the interventions Reuland et al., Owens et al., and Ruparel et al. developed [24,43,46]. Telehealth interventions, like those created by Begnaud et al. and Jansen et al., further increased accessibility by allowing for direct scheduling of lung cancer screening through electronic patient portals [25,38]. Other interventions included interactive features. Schapira et al. developed an app that allowed veterans to communicate directly with healthcare providers to reinforce educational content [41]. Integration with electronic medical records also streamlined participants’ engagement with screening promotion material [28,30,38,39,49]. Stang et al. developed an Epic-based intervention that automated lung cancer screening scheduling by forwarding patients’ replies to Epic messages to healthcare providers and simultaneously putting in an order for lung cancer screening [28]. Similarly, the tool Kukhareva et al. developed used EHR-based clinical reminders to promote lung cancer screening, effectively closing care gaps between patients and healthcare providers [49].

#### 3.2.2. Role of Technology in Supporting Lung Cancer Screening

The technologies described in the included articles served a variety of roles. Some increased awareness of the harms and benefits of lung cancer screening [22,24,26,27,28,29,30,31,32,33,34,35,36,37,38,40,41,42,45,47,49]. Others aided study participants in making informed decisions about their screening options [22,23,26,27,29,30,33,34,36,41,43,44,46,47,48,49] and calculated personalized risk [22,30,37,42,49]. Lastly, some interventions included smoking cessation resources and counseling for lung cancer screening-eligible patients [26,27,28,29,31,32,33,41].

##### Awareness Building and Education

Various technologies educated patients and increased awareness of lung cancer, lung cancer screening, and the harms and benefits of lung cancer screening [31,40,41,42,45,47]. Volk et al., who created a video-based decision aid, assessed smokers’ perceived awareness and understanding of the advantages and disadvantages and harms and benefits of lung cancer screening using the Decisional Conflict Scale (DCS) [40]. They found a significant difference between the decision aid group and the standard education group (*p* < 0.0001) where the mean Decisional Conflict Scale informed subscale score for the intervention group was 27.1 (SD = 25.9) compared to 42.1 (SD = 30.8) (DCS on a scale of 0 to 100 with lower scores indicating lower decisional conflict and increased awareness) [40]. Schapira et al. developed LCSDecTool, which educated patients about the benefits and harms of lung cancer screening [29]. LCSDecTool showed a significant difference in lung cancer screening uptake at both the 6-month and 9-month follow-up between the experimental arm (37.7% at 6 months, 44.9% at 9 months) and the control arm (21.1% at 6 months, 25.4% at 9 months) (*p* = 0.04 and *p* = 0.02, respectively) [29]. Carter-Harris et al. developed LungTalk, a web-based tool that aimed to increase awareness and prepare individuals to make lung cancer screening decisions [42]. Knowledge scores among the LungTalk group increased more than the control group [mean difference of 2.33 (SD = 1.5) vs. mean difference of 1.14 (SD = 1) on a scale of 1–6]; both changes were significant (*p* < 0.01) [42]. Patients who used the video-based decision tool that Hoffman et al. developed reported feeling more informed after the intervention and scored higher on the post-decision aid survey [mean difference of 3.9 (SD = 2.9) before and after intervention] (*p* < 0.001).

Patients who used Hoffman et al.’s video-based decision tool reported a mean increase of 3.9 points (SD = 2.9) on the 10-point post-decision-aid knowledge survey, before and post-intervention (*p* < 0.001) [47]. Sharma et al. developed a telephone-based intervention that aimed to provide in-depth messaging to patients to increase their awareness of the perceived benefit, perceived barriers, and cues to action of lung cancer screening [31]. Among the participants, 9.5% underwent LDCT (18 in the control group and 23 in the intervention group), but the difference in lung cancer screening uptake between arms was not significant (*p* = 0.68) [31].

##### Decision Support

Many interventions included decision support components, providing patients with the necessary resources to make informed decisions about lung cancer screening options [22,23,26,27,28,29,30,32,33,34,35,36,37,40,41,43,44,46,48,49]. Decisional conflict decreased significantly after viewing the decision aid developed by Lau et al., with a mean DCS score before viewing the decision aid of 46.33 (SD = 29.69), and a mean DCS score after viewing the decision aid of 15.08 (SD = 22.78) (*p* < 0.001), where any score less than 25 (out of 100) indicates that a patient is feeling ready to make a decision [22]. In a different web-based intervention developed by Lau et al., decisional conflict was 17.46 (SD = 11.44) before and 8.89 (SD = 9.65) after the intervention, a decrease of 49% (*p* < 0.001) [36]. Similarly, decisional conflict among patients using the tool Volk et al. developed was 7.84 (SD = 23.18) after viewing the decision aid [23]. Webster et al. found that decisional conflict between the intervention group and control group did not differ significantly [27]. Additionally, they found that screening uptake behaviors did not differ significantly between the intervention and control groups (*p* = 0.86) [27]. The tool Stang et al. developed offered SDM visits with providers for lung cancer screening [28]. Among the nine lung cancer screening-eligible patients, two completed an SDM visit and LDCT (22.2%) [28]. Dharod et al. found that 81 out of 99 (81.8%) screening-eligible patients who received their EHR-based intervention made a screening decision [30]. Ultimately, 5 out of 24 patients (6.2%) who desired to be screened completed a chest CT [30]. Raz et al. developed a web-based educational tool including a module with decision-making assistance [32]. Among the patients that received the intervention, 18.5% received LDCTs within 6 months after completing the survey, compared to 8.6% of the controls (*p* = 0.06) [32]. In a different tool developed by Volk et al., participants in the intervention group faced significantly lower decisional conflict (27.1 (SD = 25.9)) compared to the control group (42.1 (SD = 30.8)) (*p* < 0.0001) [33]. Ruparel et al. noticed a significant difference in decisional conflict between the intervention and control groups as well, reflecting more decision certainty in the decision group (*p* = 0.007) [43].

##### Smoking Cessation

Eight studies include smoking cessation resources or counseling in the lung cancer screening promotion tools [26,27,28,29,31,32,33,41]. Three of the interventions were administered through state-level tobacco quitlines themselves [27,31,32]. Schapira et al. conducted a two-phase study where they sought feedback from participants during Phase 1 and user testing during Phase 2 [29]. During Phase 1, they found that 38% of participants and 67% of clinicians wanted smoking cessation resources included in LCSDecTool [29]. Stang et al. designed their tool to initiate tobacco treatment should a patient be eligible for lung cancer screening and engage with the EHR message sent to them via the patient portal [28]. Volk et al. also emphasized smoking cessation throughout their video decision aid “Lung Cancer Screening: Is it Right for Me?” [33]. In addition, they included information about how to calculate tobacco pack-year smoking history, allowing participants to understand the importance of smoking cessation [33].

#### 3.2.3. Barriers in Technology-Based Lung Cancer Screening Promotion

Several barriers exist for patients using technology for lung cancer screening promotion, including system-level barriers, provider-level barriers, and patient-level barriers.

##### System-Level Barriers

At the system level, the topic of accessibility presents a significant barrier to technology-based lung cancer screening promotion. Technology-based interventions for lung cancer screening promotion often rely on access to computers or mobile devices, and a lack of access to computers or mobile devices poses a challenge for some individuals, especially in low-resource settings [22,27,33,36]. Lau et al. discuss how their web-based decision aid requires patients to have access to a computer [22]. Similarly, the video-based intervention developed by Volk et al. allowed participants to view the video on a DVD or via the internet [33]. However, if neither option were accessible (i.e., a patient did not have access to a computer or DVD player), research staff assisted participants in finding a location where they could gain local access (e.g., public libraries) [33].

Furthermore, Webster et al. discussed the issue of internet accessibility, an additional system-level barrier on top of computer and mobile device accessibility [27]. In their study, 21.1% of participants reported having no internet access at home or work, making access to their web-based intervention difficult [27]. However, those who did not have internet access randomized to the web arm of the study were then advised on other ways to view the web-based tool (e.g., using a friend or relative’s smartphone, public library) [27]. Lau et al. also highlighted the issue of internet access, with 48.6% of study participants reporting no internet access at home, and half of the study participants opting for a paper survey instead [36]. To alleviate this barrier of lack of internet access in future studies, Lau et al. suggest partnering with community organizations and conducting group learning sessions to review the decision aid [36].

##### Provider-Level Barriers

Three studies discussed the time pressure for providers to engage in a discussion about lung cancer screening, presenting as a barrier for patients looking to screen for lung cancer [46]. Owens et al. developed a study that included a shared decision-making (SDM) component. They indicated that some healthcare providers may be hesitant to engage in such a study because of their lack of time to engage in SDM and/or a lack of experience implementing SDM in their practice [46]. Similarly, Stang et al. also note insufficient time and knowledge of SDM as a barrier to lung cancer screening [28]. In addition, they cite lack of familiarity with eligibility criteria, skepticism about the benefits of screening, and familiarity with managing findings as lung cancer screening uptake barriers as well [28].

##### Patient-Level Barriers

The studies cover different patient-level barriers, such as low health literacy, psychological effects, and stigma associated with lung cancer. Patients with low health literacy may encounter challenges in comprehending online health information in comparison to those with high health literacy. In the study Lau et al. conducted, 60.8% of participants had trouble understanding written health information [36]. Similarly, Zhang et al. report a mean eHealth literacy score of 5.87 (on a scale of 0 to 8) with 43% of participants falling below the middle level [26]. High information burden can ultimately discourage individuals with low health literacy from taking part in screening [43].

Furthermore, some studies discussed psychological effects, such as heightened anxiety, associated with lung cancer screening that may deter patients from choosing to partake in lung cancer screening promotion. Reuland et al. included in their intervention information about potential psychological side effects, like anxiety and distress, but their analysis did not assess such effects post-intervention [24]. In their analysis, Schapira et al. included anxiety as a variable in their regression. Still, they found there was no difference in anxiety-level changes between the experimental and control groups (*p* = 0.84 immediately after intervention, *p* = 0.34 at 1-month follow-up, *p* = 0.74 at 3-month follow-up) [29]. Ultimately, anxiety can serve as a potential deterrent from lung cancer screening and has been shown to be associated with decreased intention to screen; technology-based lung cancer screening promotion tools, especially those that emphasize patient education, have the potential to allow patients to weigh such factors and make decisions in line with their values and needs [30,35].

##### Screening-Specific Barriers

In general, many study participants are apprehensive about screening for lung cancer because they perceive the risk of harm to be greater than the potential benefits. For example, Strong et al. describe how fear of radiation exposure can lead to a decreased intention to screen [35]. Reuland et al. also indicate that certain harms of lung cancer screening, such as overdiagnosis and increased risk of false positives, can cause substantial harms in screened populations [24].

In addition to the physical and psychological harms that lung cancer screening poses, some studies discuss screening-specific barriers, such as the cost of lung cancer screening. First, lung cancer screening can lead to costly follow-up tests and procedures [24,27,28,30,31,45]. However, lung cancer screening promotion tools can be used to weigh cost as a barrier and potentially get individuals to screen for lung cancer [30]. Webster et al. assessed study participants’ worry about cost among participants who decided not to schedule or complete lung cancer screening. They found that, in total, 6.8% of participants expressed a worry about cost: 5.0% in the intervention group, and 8.8% in the control group [27]. Sharma et al. also assessed cost in their intervention [31]. They found that, among the study participants in the intervention group, the odds of patients speaking to their insurance company about covering the cost of lung cancer screening are 1.52 times the odds of patients in the control group (95% CI: 0.93, 2.49) [31]. This demonstrates that, before the intervention, the odds of voicing cost concerns to insurance companies were lower than after the intervention, presenting a challenge for patients undergoing lung cancer screening where cost is a concern [31]. Clark et al. assessed the importance of cost for participants in their decision to screen for lung cancer or not [45]. “Avoiding out-of-pocket costs” ranked second (following “reducing the chance of death from lung cancer”) in terms of importance in participants’ decision to screen (3.7 ± 1.2, on a scale of 1–5) [45].

## 4. Discussion

This study examines the use of technology for LCS promotion. It analyzes findings from 28 studies published between 2014 and 2025. Our findings reveal a growing body of evidence leveraging digital health to address barriers to LCS, increase patient awareness, facilitate SDM, and support smoking cessation efforts. Tools ranged from videos, web-based platforms, to EHR messaging and telehealth consultations. The increasing publication trend over time highlights the rising interest in integrating eHealth within the cancer preventive realm [50].

### 4.1. Potential of Technology in Promoting Lung Cancer Screening

#### 4.1.1. Important Features for Consideration in Tool and Intervention Design

Most tools described in the included studies were specifically developed by researchers for their respective studies, while a smaller number utilized previously developed or commercially available technologies, consistent with the literature on the importance of custom-built interventions in ensuring tight alignment with the study context, population, and objectives instead of adapting off-the-shelf solutions [50]. Importantly, 70% of the tools were designed for home use, mirroring a strong emphasis on self-guided decentralized care as an effort to overcome logistical barriers such as transportation and appointment availability [50]. Tools like the visual aids and patient stories used by Volk et al. [33] have been previously shown to be powerful in reducing decisional conflict and enhancing knowledge retention [51], which may explain their potential in promoting LCS in this case [33]. In addition, personalization, seen for example in the integration of tools like pack-year calculators, was also helpful in tailoring messaging efforts based on individual risk profiles [27]. Previous research has proven that personalized interventions are more likely to result in behavioral change compared to generic content [52]. Furthermore, accessibility features such as read-aloud functions, multimedia content integration, and simplified navigation were commonly used in tools that target lower literacy levels [23,24,26,27,28,29,37,42,43,45,46]. These features remain critically important, especially since the literature has associated low screening levels with digital and health literacy levels [53]. It is also noteworthy that incorporating interactivity and integration with EMRs has the potential to enhance continuity of care and streamlining the communication between clinicians and their patients [28,30,38,39,41,49], contributing to improvements in outcome measurement as shown in the prior literature [54]. These findings suggest that developing tools for LCS promotion could be improved by prioritizing the integration of features such as (1) home-based accessibility to support uptake beyond clinic walls, (2) personalization, accounting for risk factors such as smoking history, (3) health literacy and digital literacy-sensitive designs that ensure usability among vulnerable populations, and (4) EMR integration to improve screening workflows.

#### 4.1.2. Role of Technology in Screening Promotion

**Figure 4:**
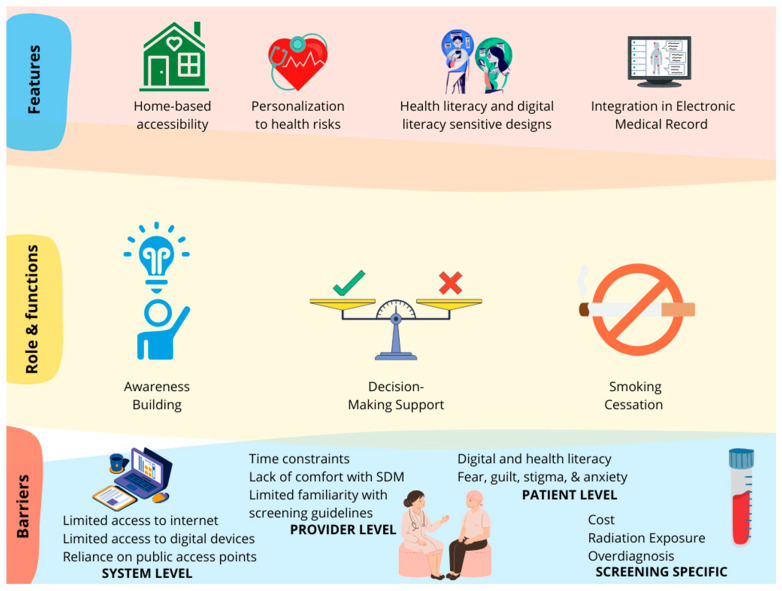
Summary of the findings.

Different tools in this study served several overlapping but distinct roles, including (1) patient awareness, (2) decision support, and in some cases (3) smoking cessation resources merged with screening promotion, as shown in Figure 4.

##### Awareness and Education

Most of the studies, particularly videos and interactive web-based platforms, focused on promoting awareness and knowledge [22,24,26,27,28,29,30,31,32,33,34,35,36,37,38,40,41,42,45,47,49], aligning with the literature linking low LCS uptake to lack of knowledge, especially among underserved populations [55]. Educational content covered screening eligibility, risk factors, and potential harms and benefits, aligning with the core principles of SDM endorsed by the US Preventive Services Task Force and the Centers for Medicare and Medicaid Services guidelines for lung cancer screening promotion [50,56]. Tools like LungTalk [42] and the decision aid developed by Volk et al. [40] were associated with significant improvements in knowledge and awareness scores. These findings are consistent with results from earlier research identifying technology as better than static print materials in improving cancer screening education [57]. These results also echo broader health communication research showing that visual and narrative-driven interventions reduce cognitive burden and improve message retention, particularly in populations with limited health literacy [3]. However, while awareness increased, some studies reported no significant impact on screening uptake [31], which could reinforce previous studies suggesting that supporting decision-making does not necessarily lead to behavioral change [58].

##### Decision Support

Among the included studies in this review, technology-based interventions used for decision-making support were the most impactful as they were able to reduce decisional conflicts [33,36]. The results were consistent with prior reviews of decision aids across cancer contexts, which have shown that structured tools reduce uncertainty, increase screening knowledge, and improve patient satisfaction [59,60,61]. Although some tools showed positive impact, some others had no statistically significant impact on decisional conflict or screening behavior [27], contrasting with the generally positive trend [59,60,61]. This inconsistency may reflect differences in intervention delivery, population characteristics, or digital literacy. Moreover, interventions that were embedded into EHR platforms and included clinical triggers (e.g., those by et al. [28]) demonstrated not only improved decision-making but also an increased likelihood of downstream actions like SDM visits and LDCT orders [28]. This confirms past work emphasizing that decision aids linked to system-level follow-up pathways are more likely to lead to tangible health outcomes [62].

##### Smoking Cessation Integration

Although fewer interventions merged screening promotion efforts with smoking cessation interventions, those that did showed meaningful integration of behavioral support into the screening conversation [28]. This reflects prior findings that lung cancer screening presents a “teachable moment” where patients are more receptive to cessation efforts, particularly when risk information is personalized [63]. It is noteworthy, though, that there was a lack of consistent integration methodology of these interventions. Earlier research has emphasized that screening interventions without cessation components may miss critical opportunities for long-term risk reduction, especially in high-risk populations [64].

These findings highlight the importance of differentiating between these three functional roles (awareness, decision-making, and smoking cessation integration) in guiding future design. Utilizing awareness promotion tools is most useful in the early stages, particularly in people with low awareness and knowledge levels. Decision support tools, when well-designed and well-integrated into the workflows, can enhance decision quality. Smoking cessation integration represents a vital mechanism for maximizing the long-term impact of screening promotion, even though it remains underutilized. Ultimately, future studies should try to combine the three functions, informing, guiding, and enabling behavior change, within a user-centered setting, which could lead to more scalable and impactful interventions.

### 4.2. Barriers to Technology-Based Lung Cancer Screening Promotion

While technology-based interventions showed potential in promoting lung cancer screening efforts, the included studies revealed consistent and multifaceted patient-level, system-level, and provider-level barriers to adoption and engagement.

#### 4.2.1. System Level

Limited access to the internet and digital devices emerged as recurring issues in several studies [27,33,36], aligning with the broader digital divide issue [65]. At the same time, some interventions resorted to offering mitigation solutions, such as offering public internet access points that could guide users and DVDs to interested people [27]. More accessible, scalable, and sustainable solutions should be explored.

#### 4.2.2. Provider Level

The most frequent provider-level barriers mentioned were time constraints, lack of comfort with SDM processes, and lack of familiarity with screening guidelines [28,46]. These results echo previous studies showing that healthcare providers tend to deprioritize lung cancer screening due to competing clinical demands and low trust in SDM delivery [66]. Without making efforts to address clinician-level obstacles, even the most effective patient-centered tools risk failing.

#### 4.2.3. Patient Level

The usability of the tools was also impacted by patient-level barriers such as low digital and health literacy [26,36], aligning with research that suggests that health interventions may unintentionally widen the disparities and digital divide if not designed for people with low literacy levels [67,68]. Other factors, such as screening-related anxiety and fear of the unknown, may also impact patients’ intention to engage [29], consistent with the prior literature that showcased fear, guilt, and stigma to be stronger deterrents than cognition, particularly in smoking populations who know that they are at higher risk of developing lung cancer [69]. Interventions that incorporate emotional framing, motivational interviewing principles, or peer narratives may be better positioned to overcome these affective barriers.

#### 4.2.4. Screening-Specific

Some of the barriers mentioned included exposure to radiation, cost, and overdiagnosis, leading patients to think that LCS might bear more harms than benefits [24,35]. This mistrust aligns with earlier findings that patients are more hesitant to undergo lung cancer screening specifically, in part due to its recent inclusion in preventive guidelines and lingering controversy about its net benefit [70]. Finally, while not always explicitly stated, many studies documented out-of-pocket costs as a common concern, even among insured participants [27,31]. This study aligns with previous large-scale trials, which found that even small costs can disproportionately deter lower-income patients [71]. Taken together, these barriers highlight that technology-based lung cancer screening efforts must be designed and deployed with explicit attention to the infrastructure on which it will be used, the seamless integration into ongoing workflows without burdening clinicians, tailoring to cultural and literacy levels, and the financial transparency regarding its cost.

### 4.3. Limitations of the Study

This study has several limitations that should be considered when interpreting the findings. First, while the review followed PRISMA guidelines and applied a comprehensive search strategy across six major databases, the included studies were predominantly conducted in high-income countries, particularly the United States and Europe. As a result, the findings may not fully generalize to low- and middle-income countries, where digital infrastructure, healthcare delivery systems, and patient preferences may differ substantially. Second, due to the significant heterogeneity across studies in terms of intervention type, study design, outcome domains, and measurement tools, we did not conduct a meta-analysis or quantitative synthesis. Instead, we employed a qualitative thematic approach, which—while appropriate for capturing the diversity and complexity of the interventions—limits the ability to estimate effect sizes or conduct subgroup comparisons. This may reduce the precision with which conclusions about intervention effectiveness can be drawn. Third, while study selection, data extraction, and quality appraisal were conducted systematically and collaboratively by both authors, the review team was relatively small. Although consensus was reached through discussion, the absence of a larger reviewer pool and the lack of formal inter-rater reliability metrics may introduce some level of subjective bias. Fourth, there may be a risk of publication bias, as the review included only peer-reviewed articles published in English. Studies with null findings or those published in other languages may have been missed, which could skew the overall portrayal of effectiveness toward more favorable outcomes. Finally, some included studies lacked detailed reporting on key variables, such as patient demographics, intervention intensity, or context-specific barriers, limiting the ability to assess equity and implementation considerations across diverse populations. Despite these limitations, this review offers a structured synthesis of current evidence on how technology-based interventions support patients undergoing lung cancer treatment and provides a foundation for future research in this critical area.

## 5. Conclusions

This study systematically reviews the literature to assess the use of technology in promoting lung cancer screening. Various technology-based tools are assessed for their feasibility and effectiveness. We found that the tools increased awareness of lung cancer screening, facilitated decision-making, and provided smoking cessation support. Such content, in tandem with various accessibility, personalization, and integrated-EMR features, proves effective in promoting lung cancer screening uptake. That said, we discuss various barriers to lung cancer screening uptake on the system level, provider level, and patient level that present unique challenges for those aiming to undergo lung cancer screening. Overcoming these barriers requires an increase in health and digital literacy on the societal level as well as an in-depth exploration of accessible and sustainable solutions to widespread technology and internet access. In addition, careful awareness of local social infrastructure is required for technology-based solutions to lung cancer screening uptake to be most successful. This review highlights the effectiveness of technology-based solutions in the promotion of lung cancer screening. It presents areas of improvement for future technology-based tools aiming to increase lung cancer screening uptake.

## Data Availability

This is a review. All data is already attached in appendices.

## Appendix A. List of Keywords Used

**Table A1:** Medical informatics-related keyword AND lung cancer keyword AND screening-related keyword with the different keywords.

Medical Informatics-Related Keywords	Lung Cancer Keywords	Screening and Treatment-Related Keywords
Ehealth OR Informatics OR technology OR “Information Technology” OR “Electronic Health Records” OR Software OR Telemedicine OR informatics OR “information technologies” OR groupware OR “group ware” OR “computer supported cooperative work” OR “computer-supported cooperative work” OR “shared workspace” OR “shared work-space” OR “shared work space” OR “information and communication technology” OR “information and communication technology” OR “information and communication technologies” OR “information and communication technologies” OR “information systems” OR “information system” OR “data system” OR “data systems” OR “informational system” OR “computer system” OR “computer systems” OR “web-based” OR “internet-based” OR “web 2.0” OR “world wideweb” OR “world-wide web” OR “user-computer” OR “humancomputer” OR “computer interface” OR “computer interfaces” OR “health information exchange” OR “health information exchanges” OR “interoperability” OR “interoperable” OR “electronic health record” OR “electronic health records” OR “electronic medical record” OR “electronic medical records” OR “electronic patient record” OR “electronic patient records” OR “patient portal” OR “patient portals” OR “personal health record” OR “personal health records” OR “personal-health record” OR “personal-health records” OR “tethered health record” OR “tethered health records” ORmhealth OR “m-health” OR “mobile health” OR “touch screen” OR touchscreen OR “smart phone” OR ehealth OR “e-health” OR “electronic health” OR “electronic intervention” OR “digital intervention” OR “electronic tool” OR “digital tool” OR “digital technology” OR “electronic technology” OR “digital technologies” OR “digital health” OR “electronic technologies” OR “e-communication” OR “eintervention” OR telecounseling OR telemonitoring OR telemedicine OR “video visit” OR “video consult” OR “video consultation” OR telehealth OR “remotemonitoring” OR “internet communication technologies” OR “internet communication tool” OR “clinical decision support” OR teleconsultation OR teleconsultations.	“Lung Cancer” OR “lung-cancer” OR “Non-Small Cell Lung Cancer” OR NSCLC OR “Small Cell Lung Cancer” OR SCLC OR “Pulmonary Neoplasms” OR “Lung Neoplasms” OR “Lung Carcinoma”	Treatment OR Therapy OR Chemotherapy OR Radiotherapy OR Targeted Therapy OR Immunotherapy OR “Adjuvant Therapy” OR “Palliative Care”

## Appendix B. Data Extraction Form

**Table A2:** Extraction Form.

Variable	Description
ID	
Title	Title of article
Year	Year of publication
Objective	Objective of the study
Study design	Observational studies; cross-sectional studies; cohort studies (prospective and retrospective); case–control studies; ecological studies (exposure and outcomes studies); descriptive studies; experimental studies; randomized controlled trials (RCT); quasi-experimental studies; community trials; qualitative studies; mixed-methods studies
Intervention	Brief description of the intervention described in the study
Who is delivering the intervention	Researchers; healthcare providers; counselors
Setting	Home-based; clinic-based; or both
Recruitment methods	Clinic-based; online-based; in-person
Length of interventions	How many minutes/days/months the intervention lasts
Frequency of interventions	One time; number of days/months/years
Country/Region	Geographical location of study
Is it designed for rural/urban/ both populations/ not specified	Rural; urban; both; not specified
Smoking composition of the population (if reported)	Percentage of current smokers; percentage of former smokers; percentage of non-smokers
Challenges of screening addressed	Challenges of lung cancer screening addressed in the article
Type of technology	The mode of technology through which the intervention was delivered (e.g., website, video, EHR message)
Purpose of technology	Awareness building; decision-making support; risk assessment; appointment scheduling; education or behavior change intervention; other
Features of technology	Personalization (e.g., tailored reminders, recommendations); Interactivity level (e.g., static vs. dynamic content, gamification, interactive modules, AI-driven recommendations); accessibility (e.g., languages offered, availability for low-literacy users); integration with healthcare systems (e.g., EHR compatibility)
Does the tool provide smoking cessation counseling	Yes/No
Commercial tool/designed specifically for the study	Was the tool designed to be used in commercial settings or was the intervention designed specifically for the study?
Does the intervention (tool) consider any health and digital literacy	Yes/No. Was the intervention developed to a specific reading level? Were there other features that helped users comprehend the text in addition to the text itself?
For personal use or assisted use	Is the tool designed to be used with an assist from a family member/provider/other, or is the tool designed to be used independently?
Impact of technology on screening outcomes	Number of people screened after technology-based intervention (compared to number of people screened in control/before intervention)
Challenges of technology use	Challenges of technology described in the articles (e.g., computer/smartphone/internet access, font size, privacy concerns)
Challenge theme (to cluster later)	
Outcomes (measures)	Screening uptake (e.g., percentage of participants completing screenings); decision-making quality (e.g., decisional conflict, patient satisfaction, informed decision-making scores); awareness levels (e.g., change in knowledge, misconceptions corrected, understanding of risk factors); behavioral changes (e.g., smoking cessation, adherence to follow-up care, LCS uptake, engagement in decision-making, etc.); health outcomes (e.g., early-stage cancer detection rates, reduction in advanced cancer diagnoses, mortality rate, etc.); engagement metrics (e.g., frequency of technology use, completion rates of modules); patient empowerment (e.g., perceived control, self-efficacy)
Outcomes (findings)	Quantified data that support outcome measures
Study limitations	Limitations of the study described by the article
What was the technology compared to	Paper-based materials; standard education materials; other web-based interventions
What were the findings compared to the traditional or other modes of interventions	How did the technology-based intervention compare to the control?
What were the barriers to implementation	BARRIERS: Technical barriers (e.g., device limitations, connectivity issues); patient-level barriers (e.g., digital literacy, cultural stigma); system and environmental barriers (e.g., impact of friends on decision-making) FACILITATORS: Stakeholder involvement (e.g., healthcare providers, community outreach); training or education provided to patients
Community and cultural relevance of the technology	Technology tailored to specific cultural needs; inclusion of underserved populations (e.g., low-income, rural communities)

## Appendix C. Table of Findings

**Table A3:** Results of the data extraction.

ID	Title	Year	Objective	Study Design	Intervention	Delivery Personnel	Setting	Recruitment Methods	Length of Interventions	Intervention Frequency	Country/Region	Designed for Rural/Urban/both Populations	Smoking Composition of the Population	Challenges of Screening Addressed
[22]	Evaluation of a Personalized, Web-Based Decision Aid for Lung Cancer Screening	2015	Assess the efficacy of a web-based patient decision aid for lung cancer screening	Uncontrolled, before-and-after study	Web-based decision aid	Research staff	Home-based	Convenience sample of volunteers (N = 60)	~10 min	One time	United States	Not specified	Mean 24.08 pack-year smoking history; N = 60 current or former smokers	Follow-up diagnostic testing; overdiagnosis; false positive rate; total radiation exposure
[23]	Feasibility of a Patient Decision Aid about Lung Cancer Screening with Low-dose Computed Tomography	2014	Development and testing of a brief, video-based patient decision aid about lung cancer screening	Uncontrolled, before-and-after design	Video-based patient decision aid	Research staff	Clinic-based	Patients from a tobacco treatment program at a large cancer center	6 min video	One time	United States	Not specified	Mean 30 pack-year smoking history; N = 23 (44.2%) current smokers, N = 29 (55.8%) former smokers	Radiation exposure; high false-positive rate with invasive testing a follow-up; overtreatment of nonfatal cancers; psychological harms (anxiety, depression); real or perceived financial strain
[24]	A pre–post study testing a lung cancer screening decision aid in primary care	2018	Testing of a lung cancer screening decision aid video in screening-eligible primary care patients	Single-group (screening-eligible), before-and-after study	Video decision aid	Research staff	Clinic-based—academic internal medicine practice	Mail and online; primary providers approved list of patients; patients mailed recruitment packet (study invitation letter, opt-out card); patients received recruitment phone call (eligibility survey); participants received a USD 40 gift card	6 min video	One time	United States	Not specified	Mean 52 pack-year smoking history; 46% current smokers (avg 52 pack-years smoked), 54% former smokers	Costly and invasive follow-up; overdiagnosis; lack of LCS knowledge
[25]	Population health nurse-driven lung cancer screening	2024	Pilot of a patient-centered population health nurse-driven LCS intervention in a general internal medicine practice embedded in an urban safety-net health system	Pilot study	Telemedicine visit with population health nurse	Research staff	Home-based	Phone-based patients with the greatest number of overdue screenings are contacted and scheduled for a telehealth visit where the nurse performs lung cancer screening opportunities	Not specified	One time	United States	Not specified	N = 237 patients with current or previous smoking history	Competing priorities in the medical visit; lack of documentation of robust smoking histories; absence of clinical decision support for primary care teams; patient and provider knowledge gaps; access to screening
[26]	“I Saw it Incidentally but Frequently”: Exploring the Effects of Online Health Information Scanning on Lung Cancer Screening Behaviors Among Chinese Smokers	2024	Investigate whether online health information scanning can effectively encourage lung cancer screening and elucidate the mechanisms driving this association among smokers	Random-sample survey	Online health information - News apps on smartphones - Health-related apps on smartphones - Social media - Search engines	Research staff	Home-based	Online from Kantar’s survey panel	Not specified	Variable health information seeking measured frequency participants searched for health information	China	Not specified	N = 992 individuals who have cumulatively smoked 100 cigarettes and have reported cigarette use in the last 30 days	Not specified
[27]	The tobacco quitline setting as a teachable moment: The Educating Quitline Users About Lung (EQUAL) cancer screening randomized trial	2023	Assess if state-based tobacco quitlines serve as a teachable moment for LCS-eligible individuals	Randomized control trial	Web-based decision tool	Research staff	Home-based	Individuals seeking phone-based cessation treatment from the Maryland Tobacco Quitline	~15 min	One time	United States	Not specified	Mean 60.3 pack-year smoking history; 70.1% currently smoking	Time (too busy, not enough time); low priority (concurrent health issues); logistics (transportation issues); worry (worried about cost, worry that screening will find something wrong, worried the scan will be dangerous); knowledge and attitudes (do not feel they need to be screened, not aware of LCS, want to quit smoking first); health system barriers (no insurance or PCP, PCP never brought it up)
[29]	A veteran-centric web-based decision aid for lung cancer screening: usability analysis	2022	Conduct usability testing of an LCSDecTool designed for veterans receiving care at a Veteran Affairs medical center	Usability testing	Computer-based	Research staff	Both settings	Online-based (to identify) Corporate Data Warehouse was used to identify eligible veterans; eligible participants were mailed recruitment letters	13 min (median)	One time	United States	Not specified	At least 30 pack-year smoking history; eligible veterans had a 30 pack-year hx of smoking and continued smoking within the past 15 years	False positive tests; significant incidental findings; overdiagnosis
[28]	Development of an electronic health record self-referral tool for lung cancer screening: one-group post-test study	2023	Develop and pilot an electronic health record (EHR) patient-facing self-referral tool to an established LCS program in an academic medical center	One-group post-test study	EHR-delivered engagement message, infographic, and self-referring survey	Research staff	Home-based	Online-based; MRNs identified from another ongoing quality improvement study in the MUSC LCS program	Not specified	One time	United States	Not specified	At least 20 pack-year smoking history; N = 6 (35.2%) current smokers, N = 11 (64.7%) former smokers	Provider level: lack of familiarity with eligibility criteria; insufficient time or knowledge to conduct shared decision-making; skepticism about the benefits of screening; familiarity with managing screen-detected findings Patient level: need for more awareness of screening eligibility or programs; cost concerns; insurance status; challenges in accessing imaging sites; LCS (first preventative screening to target poor health behavior) patients are less likely to have PCP or engage in screening services
[30]	The reach and feasibility of an interactive lung cancer screening decision aid delivered by patient portal	2019	Health systems could adopt population-level approaches to screening by identifying potential screening candidates from the electronic health records and reaching out to them via the patient portal. However, whether patients would read or act on sent information is unknown. We examined the feasibility of this digital health outreach strategy	Single-arm pragmatic trial	Web- and tablet-based interactive website	Research staff	Both settings	EHR algorithm identified candidates scheduled to see PCP within 4 weeks and had logged into their patient portal account within the last 90 days; patients were sent a message via the patient portal with a hyperlink containing a unique study ID	9.6 h (median time between reading the portal message and visiting the app)	One time	United States	Not specified	At least 30 pack-year smoking history; N = 186 (19%) current smokers, N = 811 (81%) former smokers	False-positive test results leading to invasive procedures and complications; scheduling; care access limitations; complexities of varying risk and benefits of screening by individual risk factors
[31]	Using a smoking cessation quitline to promote lung cancer screening	2018	Assessing whether in-depth messaging delivered via a smoking cessation quit-line results in participants: (1) speaking to their physician or (2) an insurance company regarding lung cancer screening	Randomized trial	Phone-based messaging following brochure	Research staff	Home-based	New York State Smokers Quitline; eligible participants - current smokers - 55–79 - smoking hx of at least 30 pack-years - agreed to be contacted for a 4-month follow-up - English speaking	Telephone call	One time	United States	Not specified	Mean 45 pack-year smoking history	Low awareness of guidelines
[32]	Improving utilization of lung cancer screening through incorporating a video-based educational tool into smoking cessation counseling	2020	Investigation of the effect of LCS educational information on LDCT utilization and smoking cessation in LCS-eligible patients receiving smoking cessation counseling through video-based education	Randomized control trial	Web-based educational video	Research staff	Home-based	Online-based; individuals who currently smoke who attended at least 1 smoking cessation counseling cessation at KPSC	Less than 30 min video	One time	United States	Not specified	At least 30 pack-year smoking history; N = 584 (56.9%) currently smoking, N = 442 (43.1%) previous smokers	Limited information; majority of smoking cessation services offer no education on LDCT; limited knowledge of potential benefits of LDCT
[33]	Effect of a patient decision aid on lung cancer screening decision-making by persons who smoke	2019	Examine the effect of a patient decision aid about lung cancer screening compared with a standard educational material (EDU) on decision-making outcomes among smokers	Randomized clinical trial	Video-based: “Lung Cancer Screening: Is It Right for Me?”	Research staff	Home-based	Tobacco quitline clients from 13 states - 55–77 - 30-plus pack-year smoking hx - English speaking	9.5 min narrated video	One time	United States	Not specified	Mean 48 pack-year smoking history; N = 516 (quitline patients) current or previous smokers	Radiation exposure from screening and diagnostic imaging; high false-positive rate leading to subsequent testing
[34]	Impact of a lung cancer screening counseling and shared decision-making visit	2017	Assist patients with the decision about participation in screening through a shared decision-making visit with narrated slide show	Single-arm trial	Narrated video slide show	Research staff	Clinic-based	Clinic-based; online-based PCPs identified potentially eligible patients and screening program reviewed patient’s EMR to confirm they met screening program eligibility criteria	6 min narrated video slide show	One time	United States	Not specified	Mean 53 pack-year smoking history; out of 423 total patients, 45.2% were active smokers	Minority of patients will experience benefits of LCS; all have the potential to be harmed
[35]	Using social media as a platform for increasing Knowledge of lung cancer screening in high-risk patients	2020	Explore and assess education of and motivation to discuss lung cancer screening with healthcare providers after viewing educational materials on social media	Pre-experimental, one-group pre-test and post-test design	Video-based	Research staff	Home-based	Online-based advertisement generated through Facebook	6 min video	One time	United States	Not specified	At least 30 pack-year smoking history; N = 11 (35.5%) current smokers, N = 20 (64.5%) former smokers	Lack of patient-provider discussions about LCS; uncertainty and low motivation; lack of knowledge about LCS; fatalistic beliefs; fear of radiation exposure; anxiety related to CT scans
[36]	Lung cancer screening Knowledge, perceptions, and decision making among African Americans in Detroit, Michigan	2021	Evaluate a previously developed web-based patient-facing decision aid for lung cancer screening among African Americans in Metro Detroit	Before-and-after study	Web-based, patient decision aid	Research staff	Home-based	In-person recruitment at community events on the east side of Detroit	5–10 min	One time	United States	Urban	25% of participants reported 30 pack-year smoking history; N = 51 (68.9%) current smokers, N = 23 (31.1%) former smokers	Lower education
[37]	Aiding shared decision making in lung cancer screening: two decision tools	2020	Compare two SDM decision aids (Option Grids and Shouldiscreen.com) for shared decision-making, efficacy, decision regret, and knowledge	Randomized control trial	Web-based: two websites (www.optiongrid.org and www.shouldiscreen.com)	Research staff	Home-based	Referred by physicians	Not specified	One time	United States	Urban	At least 30 pack-year smoking history; N = 240 (all patients) had a current or previous smoking hx of at least 30 pack-years	Potential harms; false positive results; complications as a result of positive screens; overdiagnosis; radiation exposure
[38]	Randomized electronic promotion of lung cancer screening: A pilot	2017	Determine the feasibility of LCS promotion and estimate the size of the population of former smokers who are eligible for LCS screening in a single health care system	Pilot study (randomized control trial?) where participants were randomly assigned to intervention and control group	EHR-portal-based electronic messages to promote LCS	Research staff	Home-based	EHR-identified patients from University of Minnesota pulmonary clinics or primary care clinics within the past 2 years	Not specified	One time	United States	Not specified	At least 30 pack-year smoking history; N = 200 former smokers randomly allocated, N = 652 assessed for eligibility	Not specified
[39]	Implementation of an electronic clinical reminder to improve rates of lung cancers screening	2014	Implement electronic clinical reminder to improve the uptake of lung cancer screening in appropriate high-risk patients	Retrospective cohort study	EHR-delivered clinical reminders	Research staff	Home-based	EHR-based (retrospective cohort study)	Not specified	One time	United States	Not specified	N = 2372 (39.9%) met screening criteria of being a current smoker or quitting within the past 15 years and had smoked more than 30 pack-years	Not specified
[40]	A Patient Decision Aid to Help Heavy Smokers Make Decisions about Lung Cancer Screening	2019	Compare decision-making outcomes about LCS among patients recruited through state-based tobacco quitlines, where patients were randomly assigned to the decision aid or to standard educational materials about LCS	Randomized controlled trial	Video decision aid	Research staff	Home-based	Referred from smoking quitline in different states	6 min video	One time	United States	Not specified	Mean 54.6 pack-year smoking history (intervention); 55.6 (control), average 43.4 years in intervention, 43.8 years in control	Misunderstanding and concerns; lack of awareness of screening; low preparedness for decision-making
[41]	Lung Cancer Screening Decision Aid Designed for a Primary Care Setting: A Randomized Clinical Trial	2023	Evaluate the impact on an LCS decision tool on the quality of decision-making and LCS uptake	Randomized controlled trial	Web-based patient- and clinical-facing LCS decision support tool	Research staff	Home-based	Identified LCS-eligible veterans from VA Medical centers in Pennsylvania, Connecticut, and Wisconsin	1.4 min for intervention group 5.2 min for control group	One time	United States	Not specified	Mean 40.5 pack-year smoking history (intervention), 45.0 (control); current smokers: 73.9% in intervention, 57.7% in control	False-positive results; overdiagnosis; radiation exposure
[43]	Impact of a Lung Cancer Screening Information Film on Informed Decision-making: A Randomized Trial	2019	Evaluate the impact of a novel information film on informed decision-making in individuals considering participating in LCS	Randomized controlled trial	Information film, booklet, short counseling with hcp	Health care providers (nurses, clinical trial staff)	Home-based	Identified from primary care records or participants of the lung screen uptake trial	10 min video	One time	United Kingdom	Not specified	Mean 38 pack-year smoking history (intervention), 35 control; median for cigarette smoking: intervention—16 cigarettes/day, control—15 cigarettes/day; number of pack-years, median: intervention 38, control 35; year smoked, median: intervention 47, control 46	High information burden that discourages people with low literacy from taking part in LCS
[42]	Computer-Tailored Decision Support Tool for Lung Cancer Screening: Community-Based Pilot Randomized Controlled Trial	2020	Estimate the effects of a computer-tailored decision support tool that meets the certification criteria of the international Patient Decision Aid Standards that will prepare individuals and support shared decision-making in lung cancer screening decisions	Randomized controlled trial	LungTalk Interactive Program ï¼ˆcomputer-tailored decision support toolï¼‰	Research staff	Home-based	Facebook targeting advertisement	20 min baseline survey; intervention length not specified	One time	United States	Not specified	Mean 48.7 pack-year smoking history; year smoked, 36.6; former smokers, 46.7%; current smokers, 53.3%	Low level of knowledge and awareness for lung cancer screening
[44]	Telephone-Based Shared Decision-making for Lung Cancer Screening in Primary Care	2020	Examine the feasibility of application of a telephone-based decision support tool via an online tool for promoting lung cancer screening	Feasibility study	Decision Counseling ProgramÂ© (DCP) online with telephone-based call with counselor	Trained decision counselor	Home-based	Identified from electronic medical record data and recruited from calls	10–15 min baseline survey; total intervention length not specified	One time	United States	Not specified	Mean 46.54 pack-year smoking history (total), 55.0 (screened), 42.97 (not screened); current smokers, 67.9%; former smokers, 32.1%	How to effectively promote lung cancer screening rate; poor shared decision-making in clinical setting
[45]	What is the effect of a decision aid on Knowledge, values and preferences for lung cancer screening? An online pre–post study	2021	Examine whether a decision aid improves knowledge of lung cancer screening benefits and harms and which benefits and harms are most valued	Observational studies	Web-based lung cancer screening video decision aid	Not specified	Home-based	Online participant panel	3.5 min video	One time	United States	Not specified	Mean 47.2 pack-year smoking history (current smokers), 63.1 (former smokers); mean pack-year smoking for current smokers, 47.2, and mean pack-year smoking for former smokers, 63.1	Low level of knowledge for lung cancer screening
[46]	Development and testing of “Is Lung Cancer Screening for You?” A computer-based decision aid	2023	Investigate the feasibility, acceptability, usability, and preliminary effectiveness of a computer-based decision aid	Observational studies	Decision aid for lung cancer screening on RedCap	Research Staff	Clinic-based	Recruited from upcoming patients, emailing, and flyer distribution	5.95 min	One time	United States	Not specified	All current or former smokers (N = 33)	Providers have limited time and capacity to engage in shared decision-making for LCS; lack of knowledge about risk; fear; reservation because of stigma associated with lung cancer
[47]	Using a Patient Decision Aid Video to Assess Current and Former Smokers’ Values About the Harms and Benefits of Lung Cancer Screening With Low-Dose Computed Tomography	2018	Explore how current and former smokers value potential benefits and harms after watching a patient decision aid	Observational studies	Decision aid video: Lung Cancer Screening: Is It Right for Me?	Not specified	Home-based	Recruited from the tobacco treatment program at MD Anderson Center	Not specified	One time	United States	Not specified	Mean 30.4 pack-year smoking history	Low level of knowledge and awareness of LCS
[48]	Primary care outreach and decision counseling for lung cancer screening	2024	Determine the effect of an SDM intervention on lung cancer screening in primary care	Single-arm clinical trial	Decision Counseling Program, an online software that facilitate shared decision-making	Trained decision counselor	Home-based	Identified through electronic health records and recruited by mail and phone calls	10–15 min	One time	United States	Not specified	Mean 45.6 pack-year smoking history	Lack of effective methods to facilitate and integrate SDM; low lung cancer screening uptake; time pressure during primary care visits
[49]	Lung Cancer Screening Before and After a Multifaceted Electronic Health Record Intervention: A Nonrandomized Controlled Trial	2024	Assess the association of a multifaceted clinical decision support intervention with rates of identification and completion of recommended LCS-related services	Controlled interrupted time series	An EHR-integrated shared decision-making tool and narrative guidance and clinician/patient-facing reminders	Research staff	Both settings (Phase 1, clinical-based Phase 2)	Identified through electronic healthcare records	Period of 1–11 months; period of 2–9 months	Provided at each visit	United States	Not specified	Median time smoked 40 years, cigarettes per day 20, current smoker: baseline—49.8%, period 1—51.6%, period 2 51.4%.	Low LCS uptake; methods of screening other than chest CT remain unexplored
